# A Text-Book of Practical Therapeutics

**Published:** 1899-11

**Authors:** 


A Text-Book of Practical Therapeutics. With especial reference to the Application of Remedial Measures to Disease and their Employment upon a Rational Basis. By Hobart Amory Hare, M. D., Professor of Therapeutics and Materia Medica in the Jefferson Medical College, Philadelphia. With special chapters by Drs. G. E. DeSchweinitz, Edward Martin and Barton C. Hirst. New (seventh) edition. In one octavo volume of 770 pages, illustrated. Cloth, $3.75; leather, $4.50, net. Lea Brothers & Co., Philadelphia and New York.

This work has averaged a new edition a year since it first came out in 1891. A thorough revision has been made in each edition, and all of the latest real advances have been given in a comprehensive essay style. The great favor this book has received is owing to its ingenious plan and to masterful execution.

The volume consists essentially of two parts, one on drugs and other remedial measures, and the other on diseases, each being arranged alphabetically for convenience of reference. The rational classification of drugs is given succinctly in a few pages. Each of the two parts is written with the other in view, and copiously cross-referenced. The reader desiring to know all the material facts concerning a drug can instantly find the information in the first half of the book, with references carrying him to the applications in the various diseases discussed in the second part; or if desiring knowledge concerning the treatment of any special disease, he can quickly find it under the alphabet of diseases, with full therapeutical directions and prescriptions for various stages and complications, and references to the first part, where all details concerning the individual drugs are given. To consummate quickness of reference Dr. Hare has prepared two indices, likewise unique, one of drugs, and the other of diseases and remedies, the latter being annotated with suggestions, doses and other information of the most useful kind. In a word, Dr. Hare's work is not only an unrivalled text-book on therapeutics, but obviously a most convenient as well as authoritative guide to the practice of medicine. B.

